# High spatial resolution dosimetry for radiation oncology with “MagicPlates,” a new 976‐pixel monolithic silicon detector

**DOI:** 10.1002/acm2.70015

**Published:** 2025-03-13

**Authors:** Ilia Filipev, Saree Alnaghy, Martin Carolan, Jason Paino, Marco Petasecca, Dean Cutajar, Joel Poder, Justin B. Davies, Bradley M. Oborn, Nicholas Hardcastle, Susanna Guatelli, Michael Lerch, Tomas Kron, Anatoly Rosenfeld

**Affiliations:** ^1^ Centre for Medical Radiation Physics University of Wollongong New South Wales Australia; ^2^ Nelune Comprehensive Cancer Centre Prince of Wales Hospital New South Wales Australia; ^3^ Illawarra Cancer Care Centre Illawarra Shoalhaven Local Health District Wollongong New South Wales Australia; ^4^ St George Hospital Cancer Care Centre Kogarah New South Wales Australia; ^5^ Australian Nuclear Science and Technology Organisation Lucas Heights New South Wales Australia; ^6^ Helmholtz‐Zentrum Dresden‐Rossendorf Institute of Radiooncology‐OncoRay Dresden Germany; ^7^ Sir Peter MacCallum Department of Oncology University of Melbourne Melbourne Victoria Australia; ^8^ Department of Physical Sciences Peter MacCallum Cancer Centre Melbourne Victoria Australia

**Keywords:** high spatial resolution, monolithic 2D array, silicon pixelated detector, small field dosimetry, stereotactic radiotherapy

## Abstract

**Purpose:**

We introduce the next generation of “MagicPlate” 2D monolithic pixelated semiconductor detectors – MagicPlate‐976 (MP976). It features a larger array area, higher spatial resolution, and does not require external triggering. We perform a comprehensive characterization for small‐field steep‐dose‐gradient dosimetry applications in radiation therapy focusing on x‐ray beams used in stereotactic treatments.

**Methods:**

The MP976, developed by the Centre for Medical Radiation Physics, consists of 976 ion‐implanted diodes on a thin n‐type epitaxial silicon substrate with a total array area of 58 × 58 mm^2^. The central region has “small” diodes with an area of 0.2 × 0.2 mm^2^ and 1 mm pitch and the peripheral region has “large” diodes with an area of 0.6 × 0.6 mm^2^ and 2 mm pitch. The detector was primed with 10 kGy (Co‐60) and tested using a Varian TrueBeam linear accelerator for sensitivity change and dose linearity, and variations in response due to dose‐per‐pulse and beam incidence angle. Output factors, depth dose, and beam profiles were measured and compared with reference data.

**Results:**

After the 10 kGy, the sensitivity declined by (74 ± 5)% for “large” diodes and by (78 ± 7)% for the “small” ones, the dose‐per‐pulse (DPP) dependence was in the range of commercially available diodes, however, a difference in the DPP dependence between the “large” and “small” diodes of (8.4 ± 0.2)% was found in the studied DPP range from 0.131–1.111 mGy/pulse. The minimum angular response was at 90° for 6 MV and 100° for 10 MV flattened beams (76% and 82%, respectively). The output factors and depth dose response showed agreement with the reference within 3.1% and 1%, respectively. Deviation in small field 80%/20% penumbra measurements was within 0.5 mm for 6 MV FF and 0.3 mm for 10 MV FFF. Full width at half maximum (FWHM) for the beam profiles agreed within 0.5 mm for both beam qualities.

**Conclusion:**

The new MagicPlate‐976 detector system is shown to be suitable for dosimetry in small fields and steep dose gradients. It provides 1 mm spatial resolution in the central region and 2 mm on the periphery and has no dependence on the field size. The system's high spatial and temporal resolution opens new opportunities for trigger‐less, film‐less, and time‐resolved verification and error identification for complex stereotactic treatment plans.

## 1 INTRODUCTION

In radiation oncology, highly conformal hypo‐fractionated treatments with x‐ray beams, like stereotactic body radiation therapy (SBRT or stereotactic ablative body radiotherapy, SABR), deliver up to 28 Gy to a target^1^ in one fraction with steep dose gradients using small fields and often dynamic treatment modalities (i.e., volumetric modulated arc therapy or VMAT). Due to the high doses per fraction and narrow margins for error, the role of a thorough patient‐specific quality assurance (PSQA) verification with appropriate dosimetric tools increases. The ideal dosimeter would provide a 2D or 3D dose distribution, introduce no perturbation into the radiation field, have a real‐time response with temporal resolution allowing for verification of dynamic deliveries, spatial resolution of at least 2.5 mm^2^, ^3^ overall and 1 mm for high‐gradient regions (to match gamma analysis with distance to agreement of 1 mm for spinal SABR^4^), have stable dose response throughout at least one treatment delivery, be dose rate/dose per pulse, field size, energy, and beam incidence angle independent. None of the current technologies can fulfil all the criteria, however, semiconductor monolithic pixelated detectors were shown to be promising candidates.^5^

Monolithic semiconductor detector technology provides accurate control for the size, pitch, and placement of the ion‐implanted diodes, allowing for higher spatial resolution, minimizing dose volume averaging by each diode (by creating very small sensitive volumes (SV), and tailoring the detector's sensitivity. The Centre for Medical Radiation Physics (CMRP) at the University of Wollongong, Australia, has developed and successfully tested a series of 2D monolithic pixelated detectors under the MagicPlate family for various applications in radiation oncology.^6^, ^7^, ^8^ The detectors were based on a p‐type thin silicon substrate (epitaxial or bulk) with ion‐implanted diodes and a total sensitive area of up to 52 × 52 mm^2^ (MagicPlate‐512). The technological approach was proven to be successful in small‐field and high‐resolution dosimetry for clinical beams and dynamic modalities (including motion adaptive),^9^, ^10^, ^11^ however, had its limitations related to the necessary trade between the total sensitive area, spatial resolution, and the number of readout channels. Additionally, the used 64‐channel analog front end (AFE) based readout electronics^12^ required synchronization with pulsed radiation beams which complicated measurements with LINACs models and/or treatment modalities (like VMAT) that utilize variable beam pulse rates during the treatment.

In this study, we introduce the next step in the development of “MagicPlate” technology for high spatial resolution dosimetry, the MagicPlate‐976 (MP976) detector system. Compared to its predecessors, MP976 has a non‐uniform diode arrangement allowing for higher spatial resolution dosimetry (down to 1 mm pitch in the center) on a larger total sensitive area. The detector is readout with new and more compact electronics that do not require external triggering thus making it decoupled from the LINAC pulse rate. In the present work, we comprehensively characterize the MP976 for high spatial resolution dosimetry applications in radiation therapy under clinical Megavoltage x‐ray beams, focusing on the challenging quality assurance case for SABR treatments. This requires dosimetry of small radiation fields and steep dose gradients, stability over relatively large dose ranges (up to 30 Gy in the target per fraction), irradiation from different gantry angles with changing beam aperture and varying LINAC pulse rate (nominal dose rate). The present work constitutes an essential step in the clinical application of a dosimetry system to independently describe the device's performance before transitioning to more complex treatment plans and scenarios. Testing of the MP976‐based detector‐phantom system under real patient vertebral SABR plans is ongoing and is the subject of our next publications.

## 2 MATERIALS AND METHODS

### 2.1 Detector

**FIGURE 1:**
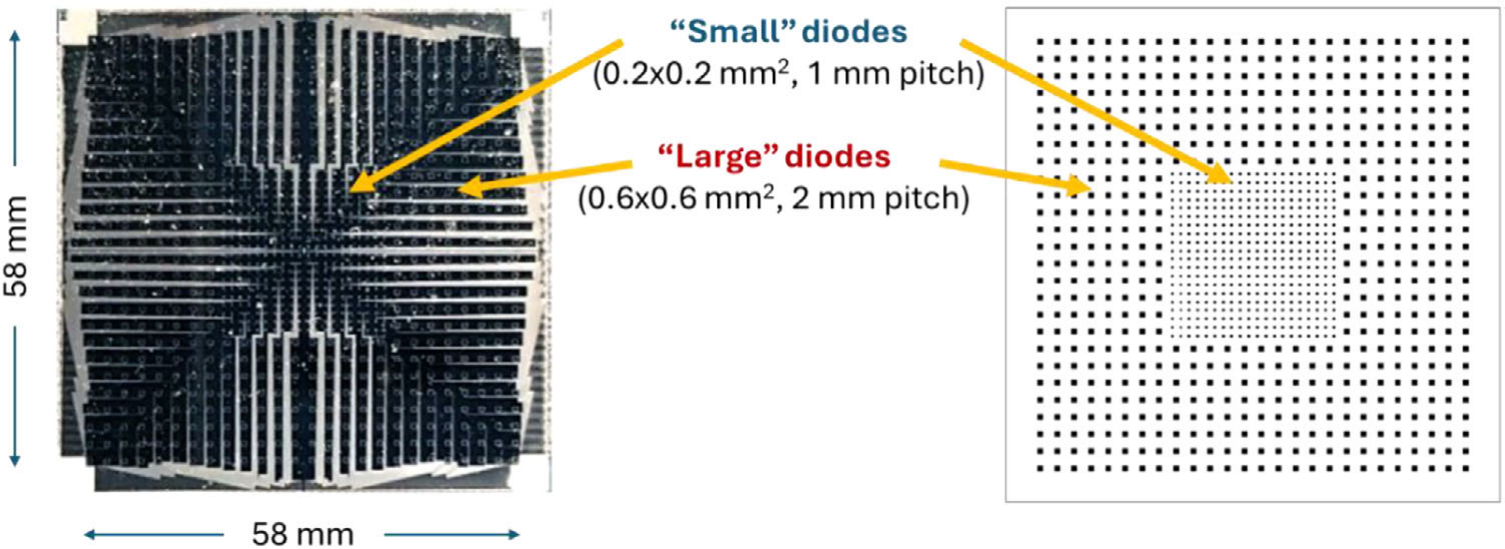
Structure of the diodes (sensitive volumes) arrangement of the MP976 detector. (left side) Photo of the detector, (right side) visualization of the diodes pattern.

MagicPlate‐976 is a 2D monolithic silicon array dosimeter with a total area of 58 × 58 mm^2^. It consists of 976 ion‐implanted planar diodes fabricated on n‐type epitaxial layer, grown on top of a low resistivity n+ substrate. The diodes are operated in a passive mode (no bias applied to the p+n junction). The detector has a non‐uniform arrangement of diodes (pitch and size) to provide superior spatial resolution in the central region while maintaining a reasonable number of readout channels. The central region (21 × 21 mm^2^) consists of “small” diodes with area of 0.2 × 0.2 mm^2^ (SV of 0.0016 mm^3^) and 1 mm pitch. The peripheral region consists of “large” diodes with an area of 0.6 × 0.6 mm^2^ (SV of 0.0144 mm^3^) and 2 mm pitch (Figure 1). The silicon detector array is wire bonded to a thin (0.5 mm thick) and flexible printed circuit board (PCB) and the detector together with the bonding wires is covered by a thin layer of protective epoxy to minimize accidental damage.

The DAQ was developed by CMRP and based on the multichannel charge‐to‐digital converter DDC264 (Texas Instruments).^12^ It has 1024 channels and is controlled by a custom‐designed field programmable gate array (FPGA) master board communicating with the PC through a USB 2.0 link. The system does not require external triggering and has real‐time readout with a sampling rate of up to 5 kHz (integration time down to 0.2 ms) and zero dead time. All the readout electronics are mounted on the same PCB with the detector for compactness and mobility of the system.

For the measurements in this work, the integration time was set to 2.8 or 10 ms depending on the required sensitivity (number of integrated LINAC pulses). Throughout each test, the integration time was kept constant.

### 2.2 Detector packaging and phantoms

**FIGURE 2:**
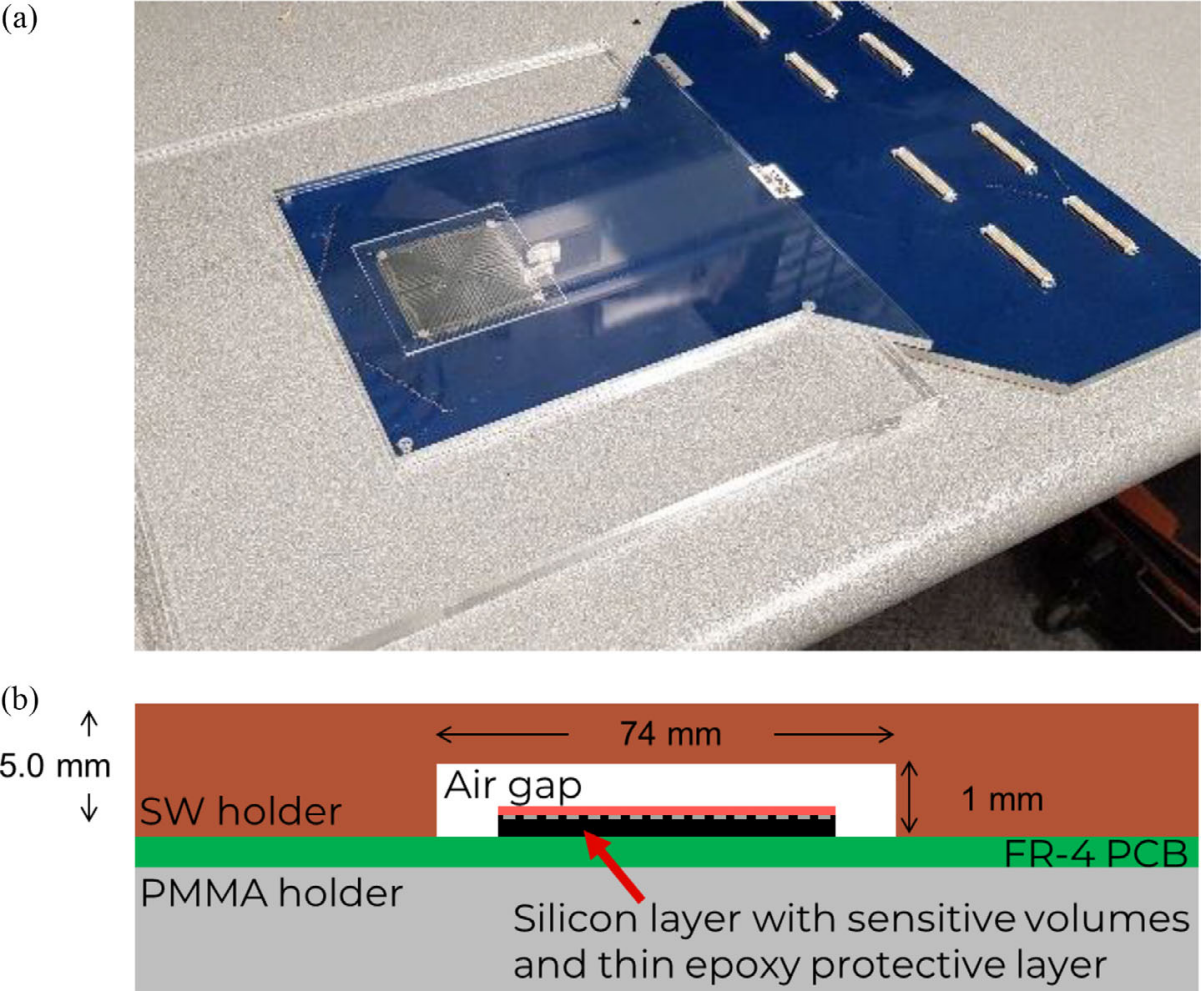
(a) MP976 detector sandwiched between polymethyl methacrylate (PMMA) holder plates and inserted into a 30 × 30 cm^2^ PMMA adapter. In all experiments, the top part of the holder was made of solid water material; in this photo, the top part is made of transparent PMMA for better visual representation. (b) Schematic representation of the MP976 detector packaging (not to scale).

During each measurement and the priming, MP976 was sandwiched between two flat pieces of water‐equivalent plastic holders, 5 mm each: RW3 solid water above the detector (with an air gap on top of the sensitive volumes) and polymethyl methacrylate (PMMA) underneath the detector (Figure 2). The air gap was introduced to compensate for the overresponse of silicon diodes in small fields and avoid output factor corrections allowing the response to be field‐size independent.^8^, ^13^, ^14^

**FIGURE 3:**
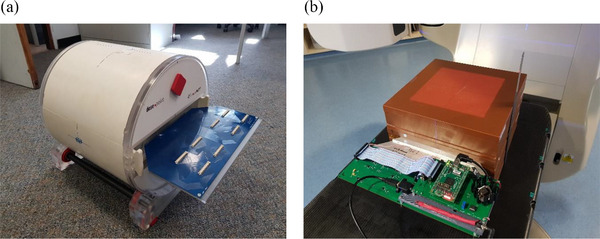
(a) DosePoint RT‐smartIMRT RW3‐based phantom used for angular dependence study. (b) MP976 sandwiched between 30 × 30 cm^2^ solid water slabs of various thicknesses.

For the priming in radiation hardness studies, additional 2 mm solid water (SW) layers were added on both sides of the “sandwich” to boost the dose from secondary electrons (7 mm in total). For the angular dependence measurements, the “sandwich” was lodged into a DosePoint (DosePoint GmBH, Wiesloch, Germany) RT‐smartIMRT RW3‐based phantom (Figure 3a). For all other measurements, 30 × 30 cm^2^ solid water slabs of various thicknesses were used to create the required build‐up and back‐scatter layers (Figure 3b) which are described in detail below.

### 2.3 Facilities

The radiation priming of MP976 was done with a Co–60 gamma source at the Gamma Technology Research Irradiator Facility (ANSTO, Lucas Heights, NSW, Australia). All other studies were performed at the Illawarra Cancer Care Centre (Wollongong, NSW, Australia) using a Varian TrueBeam (Varian Medical Systems, Palo Alto, CA, USA) LINAC calibrated to deliver 1 cGy per monitor unit (cGy/MU) at D_max_ in water at 100 cm source‐to‐surface distance (SSD). The X‐ray beam qualities used are described below.

### 2.4 Performance tests

#### 2.4.1 Radiation priming and sensitivity change with dose

To establish the change of the detector's sensitivity with accumulated dose, a series of identical measurements were performed using the TrueBeam LINAC three times: before priming, after 5 kGy priming, and after 10 kGy. Each time MP976 was placed at the LINAC isocenter perpendicular to the beam axis between solid water slabs at 10 cm depth and with 10 cm backscatter, with 100 cm SSD. Nine shots with 100 monitor units (MU) were delivered under 6 MV flattened (6X) 20 × 20 cm^2^ field and 600 MU/min dose rate (0.73 Gy each shot at the detector location). After each three shots, we delivered an additional 10 Gy of accumulated dose using the LINAC. The response was estimated separately for “large” and “small” diodes and each result represents average readings among diodes (either “large” or “small”).

#### 2.4.2 Dose linearity

A characterization of the linearity of the detector's response with the delivered dose was performed by irradiating MP976 with 6 MV flattened (600 MU/min), 10 MV flattened (10X) (600 MU/min), and 10 MV flattening‐filter‐free (10FFF) (2400 MU/min) beams with 10 × 10 cm^2^ field. The device was placed at D_max_ in solid water and with a 20 cm backscatter at 100 cm SSD. The delivered doses ranged from 20 to 3000 MU (cGy) to cover the maximum possible dose delivered in one fraction of a spinal stereotactic treatment (around 30 Gy). The measurements were performed three times each and the results represent the averaged response of the four central diodes of MP976.

#### 2.4.3 Dose‐per‐pulse dependence

High temporal resolution (the readout sampling is up to 5 kHz) and zero dead time of the MP976 system allows performing pulse‐by‐pulse dosimetry—the ability to detect individual LINAC pulses that have repetition rate in the range from 120 to 360 Hz and width of about 3–5 µs for the Varian TrueBeam accelerators. Since the nominal LINAC's dose rate (in MU/min) is determined by the variation of the pulse repetition rate without changing the dose per pulse, its variation does not create a significant difference in the MP976 response. Therefore, MP976 can be considered independent from the nominal dose rate.

**FIGURE 4:**
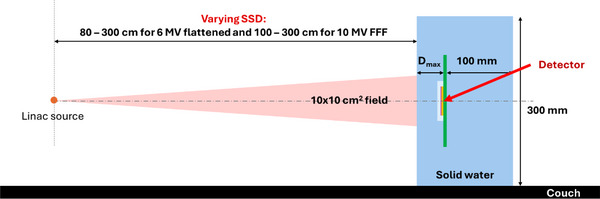
Dose‐per‐pulse (DPP) dependence study setup. MP976 detector was kept at a constant depth in solid water and the source to surface distance (SSD) was varied between 80 and 300 cm to achieve variation in the DPP. The 10 × 10 cm^2^ field size defined at the isocenter was kept unchanged.

To investigate MP976's response to variation in the dose per pulse (DPP or instantaneous dose rate) in the clinically relevant range, the detector was irradiated with a fixed number of MU and varying SSD to change the DPP at the detector's location.^15^ The DPP dependence was investigated in the range 0.032–0.432 mGy/pulse (SSD 80–300 cm) for 6 MV flattened beam and 0.131–1.111 mGy/pulse (SSD 100–300 cm) for 10 FFF beam. To achieve greater range and precision in the detector positioning, the gantry angle and the couch kick were set to 270°, and the couch longitudinal movement was used for setting various SSDs (Figure 4). MP976 was sandwiched between solid water slabs at D_max_ with a 10 cm backscatter and irradiated with 10 × 10 cm^2^ beams defined at the isocenter; the nominal doses were 50 MU with 600 MU/min for 6 MV flattened and 200 MU with 2400 MU/min for 10 MV FFF. The same measurements were repeated with the NE2571A Farmer ionization chamber (IC) as the reference and the ion‐recombination correction factor, calculated with a two‐voltage method, was applied to correct the readings of the Farmer ionization chamber.^16^

The DPP values for each SSD position were estimated using the corrected IC readings. For each diode, DPP dependence was defined as

(1) $$\mathrm{DP}P_{\mathrm{dep}}=\frac{S_{\mathrm{DPP}}}{S_{\mathrm{ref}}},$$

where the S_DPP_ is the response of the diode at any given DPP, S_ref_ is the response at DPP of 0.278 mGy/pulse (it is the DPP at 100 cm SSD, 1.5 cm depth (D_max_) for 10 × 10 cm^2^ 6X beam). The response is defined as

(2) $$S_{\mathrm{DPP}}=\frac{Q}{Q_{\mathrm{IC}}}\;,$$

where Q is the charge collected by MP976 and Q_IC_ is the charge collected by the reference IC. The DPP dependence was estimated separately for “small” and “large” diodes as an average value.

For the non‐flattened 10 MV field, the beam profile “seen” by the detector was changing with SSD due to the beam divergence. To account for this factor, only 4 central “small” diodes (2 × 2 mm^2^ area) were read out and assigned DPP values measured by the IC at the beam axis. For the “large” diodes, only one row around the central region was read out, and the assigned DPP values were estimated using the geometrical divergence of the beam profile. For each SSD, three measurements were performed and averaged.

#### 2.4.4 Output factors

The output factor (OF) measurements were performed at SSD 100 cm, detector depth of 10 cm, and square fields with sides ranging from 0.5 to 30 cm defined by the jaws at the isocenter. The beam quality was 6 MV flattened with a 600 MU/min dose rate. For each field size, three measurements with 100 MU delivery were performed and averaged. MP976 results were compared with the LINAC commissioning data (measured with CC13 chamber for fields with sides of 5–30 cm and Razor Chamber for fields of 1–4 cm (both detectors by IBA Dosimetry Louvain‐La‐Neuve, Belgium)), Monte Carlo simulations, and measurements with MO*Skin* detector performed under the same conditions. MO*Skin* is a silicon metal‐oxide‐semiconductor field effect transistor (MOSFET) detector developed by CMRP for skin dosimetry and shown^6^ to be field size independent down to 0.5 × 0.5 cm^2^. MO*Skin* measurements were performed three times and averaged.

Table 1 reports relevant parameters for the Monte Carlo simulations as recommended by the American Association of Medical Physicists (AAPM) Task Group 268 on the reporting of Monte Carlo radiation transport studies.^17^ The LINAC's multileaf collimators (MLCs) were set as retracted in each simulation to match the experimental setup.

**TABLE 1:** Summary of the main MC simulation details according to the recommendations of AAPM TG‐268.

Item	Description
Code, version/release date	EGSnrc‐2023 (BEAMnrc for the treatment head and DOSXYZnrc for the phantom dose calculations)^18^
Validation	Phase space source: Constantin et al. (2011)^19^
Timing	All simulations were performed on an in‐house Linux‐based cluster and split across 200 parallel jobs for each field size. Simulation times were proportional to the field sizes and ranged from 10 to 2000 CPU hours
Source description	The beam model uses Varian‐generated phase‐space files for each energy of the TrueBeam machine
Cross‐sections	XCOM^20^
Transport parameters	Electron‐step algorithm = PRESTA‐II. Inside the treatment head simulations (BEAMnrc) the ECUT and PCUT parameters were set at 0.521and 0.01 MeV, respectively. For the phantom simulations using DOSXYZnrc the ECUT and PCUT parameters were set at 0.7 and 0.01 MeV, respectively.
Variance reduction techniques	None
Scored quantities	Dose to medium (water). The 50 cm^3^ dose calculation phantom consisted of 2 × 2 × 2 mm^3^ voxels and was set as water with a density of 1.0 g/cm^3^
# Histories/statistical uncertainty	For each field, a total of 2 × 10^10^ primary histories were run to reduce dose calculation uncertainty to less than 2%
Postprocessing	MATLAB scripts to combine parallel job results

Abbreviation: AAMP, American Association of Medical Physicists.

#### 2.4.5 Angular dependence

**FIGURE 5:**
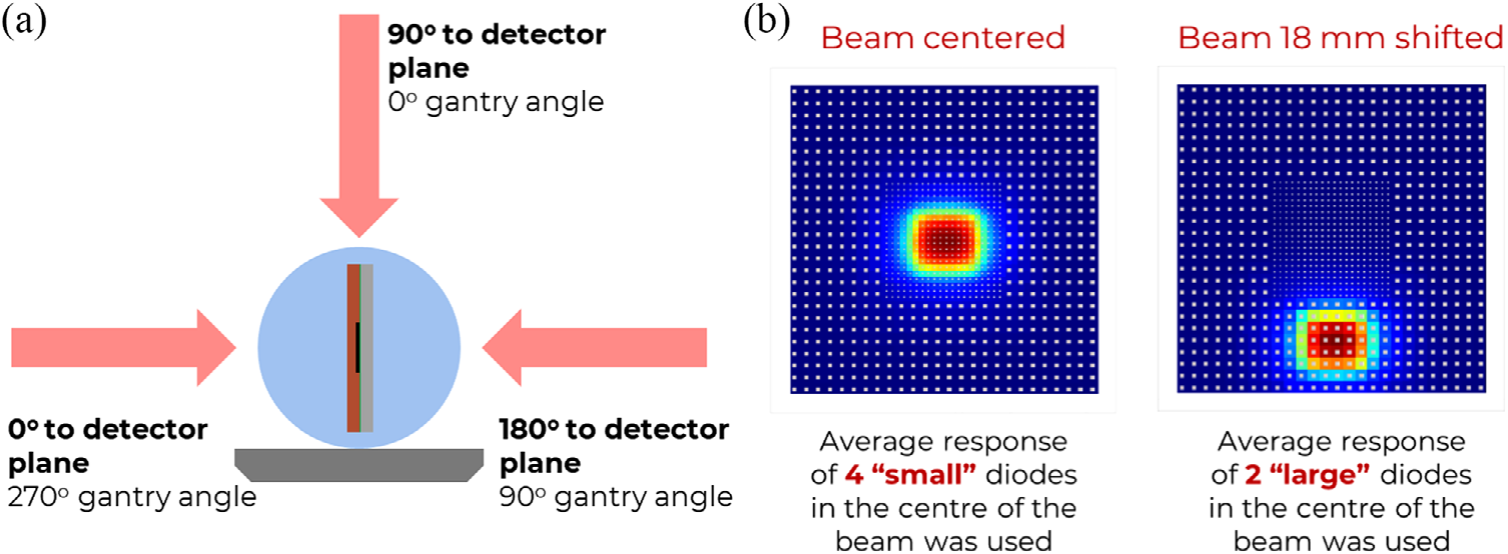
Setup for the angular dependence study. (a) The detector and beam orientation. (b) The beam position to study angular response separately for “small” (central) and “large” (peripheral) diodes; rotation of the gantry was performed around the vertical axis of the image.

For the angular dependence study, the phantom with the detector was rotated 90° to avoid irradiation through the couch (Figure 5a). The measurements were performed for 6 and 10 MV flattened beams with two jaws‐defined fields: 10 × 10 cm^2^ and 1 × 1 cm^2^. The following angles between the beam axis and the detector plane were investigated: 0°, 15°, 30°, 45°, 60°, 75°, 80°, 85°, 90°, 95°, 100°, 105°, 120°, 135°, 150°, 165°, and 180°, where 0° represent the beam being normal to the detector plane. The Centre of the detector was placed at the isocenter.

The measurements were repeated three times at each angle and the results represent the averaged response of the four central “small” diodes of MP976. To test separately the “large” diodes, the tests for 6 MV were repeated with the detector and the phantom shifted 18 mm in the sup‐inf direction (Figure 5b).

#### 2.4.6 Depth dose profile

The depth dose profiles were measured for 6X and 10FFF beams using solid water slabs with 100 cm SSD and 10 × 10 cm^2^ field. The depth was estimated based on the real thickness of the layers above the sensitive volumes (4.5 mm of the RW3 holder and epoxy resin), therefore, the following depths were used: 4.5, 9.5, 14.5, 19.5, 49.5, 99.5, and 199.5 mm. For each depth, three measurements were performed, averaged, and normalized to the response at D_max_. Based on the dose‐per‐pulse dependence study described above, the depth‐dose curves measured with MP967 were corrected for the dose per pulse.

For depths larger than D_max_, the results were compared to the LINAC commissioning data measured with CC13 ionization chamber. Results at depths smaller than D_max_ were compared to Monte Carlo simulations as the ionization chambers are not well suited for accurate measurements at shallow depths. The Monte Carlo simulations were performed under the same parameters as those used in the output factor study described above.

#### 2.4.7 Small field off‐axis profiles

In order to study MP976's ability to correctly measure penumbral region (defined as the region included between 20% and 80% of the maximum dose intensity^21^), particularly in small radiation fields, small field off‐axis (OAX) profiles were measured with the detector. MP976 was placed at 10 cm depth in solid water at 100 cm SSD with its center aligned with the beam axis and irradiated with 6X and 10FFF beams with filed sizes of 1 × 1, 2 × 2, 3 × 3, and 4 × 4 cm^2^ defined by the LINAC jaws at the isocenter (the multileaf collimator completely retracted). As the backscatter, 20 cm of solid water slabs were placed underneath the detector. In order to equalize the readings of each diode, equalization measurements were performed at the same depth by irradiating with 20 × 20 cm^2^ field size and 6X beam quality to achieve a flat field. Each field was measured three times, averaged, and normalized to the averaged response of the central four diodes of the 4 × 4 cm^2^ field measurement.

Readings of MP976 were compared with the LINAC commissioning data measured with EFD‐3G diode (Scanditronix Wellhofer AB, Uppsala, Sweden). As the MP976 detector has an even number of diodes, the OAX profiles were calculated as the average between the results of the two diode rows adjacent to the beam axis.

A quantitative comparison of the profiles was performed using two criteria: full width at half maximum (FWHM) and the 80%/20% ratio for the penumbral region. To estimate the criteria, a cubic spline interpolation of the data point was used with a resolution step of 0.01 mm. To manage noise present in several reference measurements, the data was pre‐smoothed using a Gaussian filter while keeping the shape preserved. Results for left‐hand‐side (LHS) and right‐hand‐side (RHS) penumbrae were averaged.

## 3 RESULTS

### 3.1 Radiation priming and sensitivity change with dose

**FIGURE 6:**
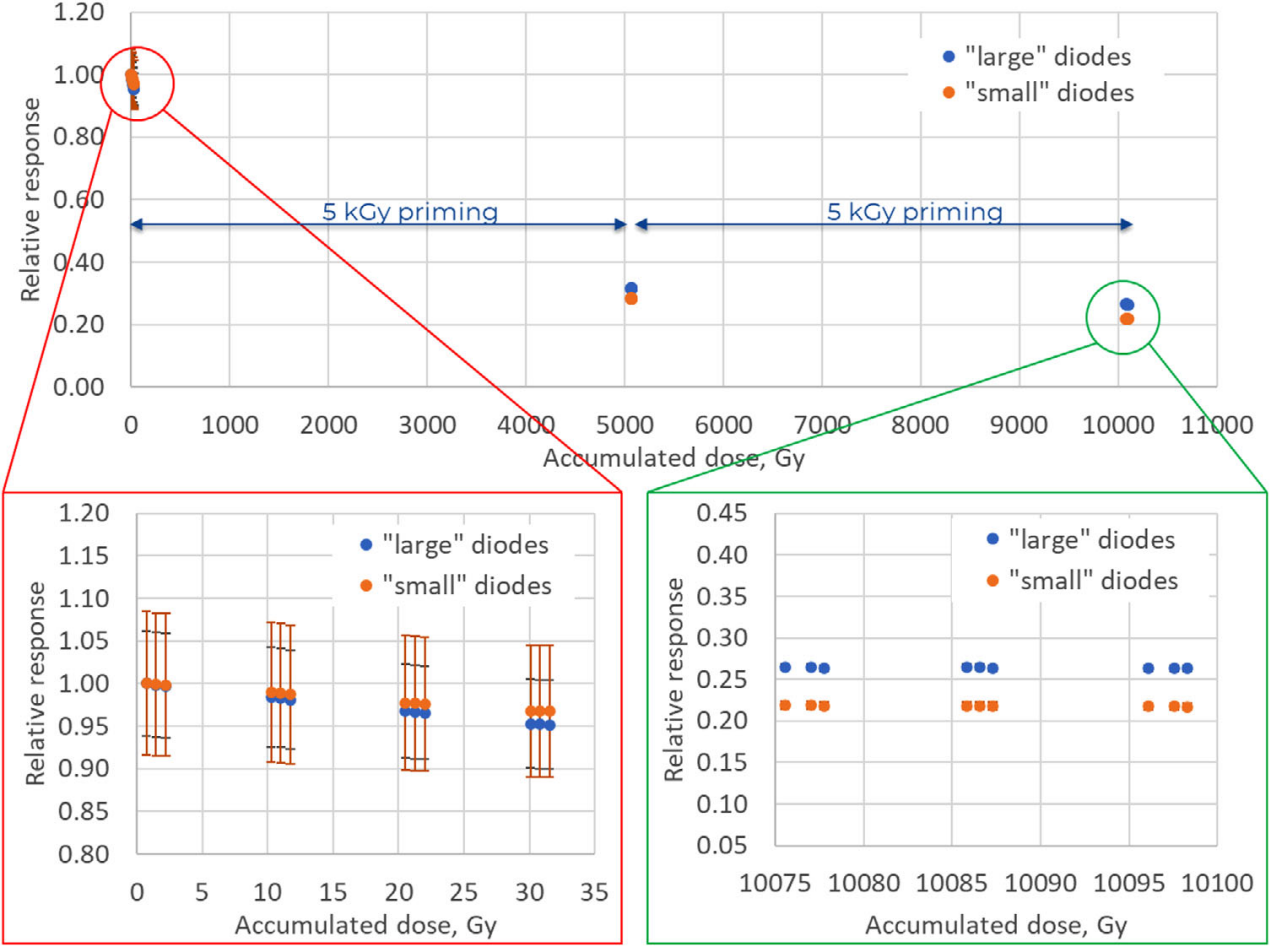
Variation of the response of MP976 as a function of the accumulated dose by irradiation with a Co‐60 gamma source. Each point represents the averaged response of diodes to 0.73 Gy delivered with 6 MV FF LINAC X‐ray beam. The error bars represent one standard deviation among diode readings.

Results for the sensitivity change with priming of the MP976 detector are depicted in Figure 6. The shown values are normalized to that at zero accumulated dose. The error bars represent one standard deviation among diode readings. After the priming, MP976 diodes achieved a low sensitivity change to perform all described performance tests. For future routine use in the clinical environment, the detector will be primed more to reach the sensitivity change values of single diodes used in commercially available arrays.^22^

A linear fit of the results after 10 kGy priming gives the sensitivity change (for 6 MV FF) of −0.016% Gy^−1^ for “large” diodes and −0.031% Gy^−1^ for “small” diodes. Overall, the sensitivity dropped by (74 ± 5)% for the “large” diodes and by (78 ± 7)% for the “small”, giving the resulting values of (201 ± 3) and (51 ± 1) pC/cGy, respectively.

### 3.2 Dose linearity

**FIGURE 7:**
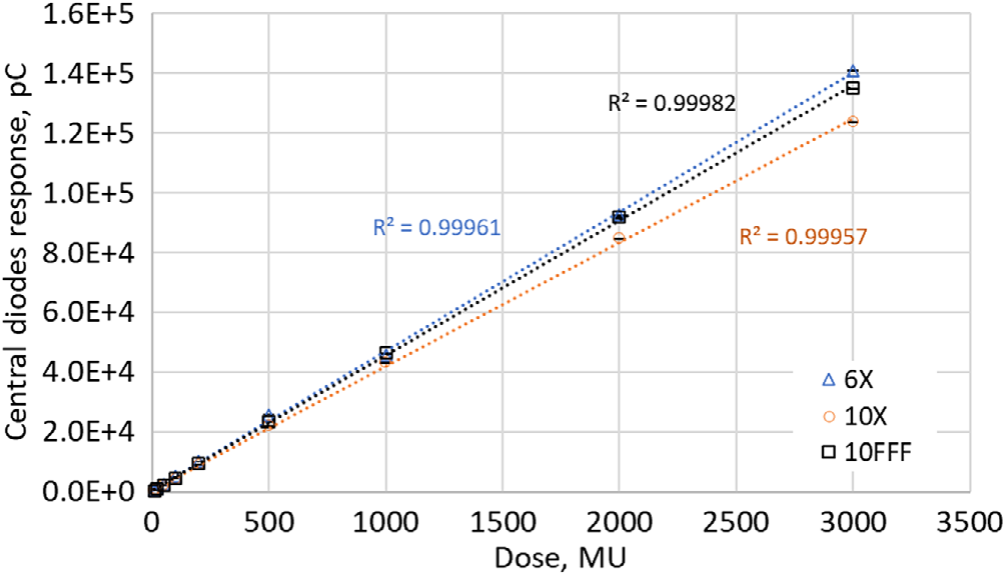
Dose linearity results for the MP976 detector, measured under 6X, 10X, and 10FFF beam qualities at D_max_, 100 cm SSD, 10 × 10 cm^2^ field size. Results represent the average of 4 central diodes. Dotted lines represent linear fit, the corresponding adjusted regression coefficients (R^2^) are stated on the plot.

Figure 7 shows the results of the study of MP976's response linearity with dose. The error bars (one standard deviation over three measurements) stay within the marker size and for all beam qualities with a maximum of 1.2%.

### 3.3 Dose‐per‐pulse dependence

**FIGURE 8:**
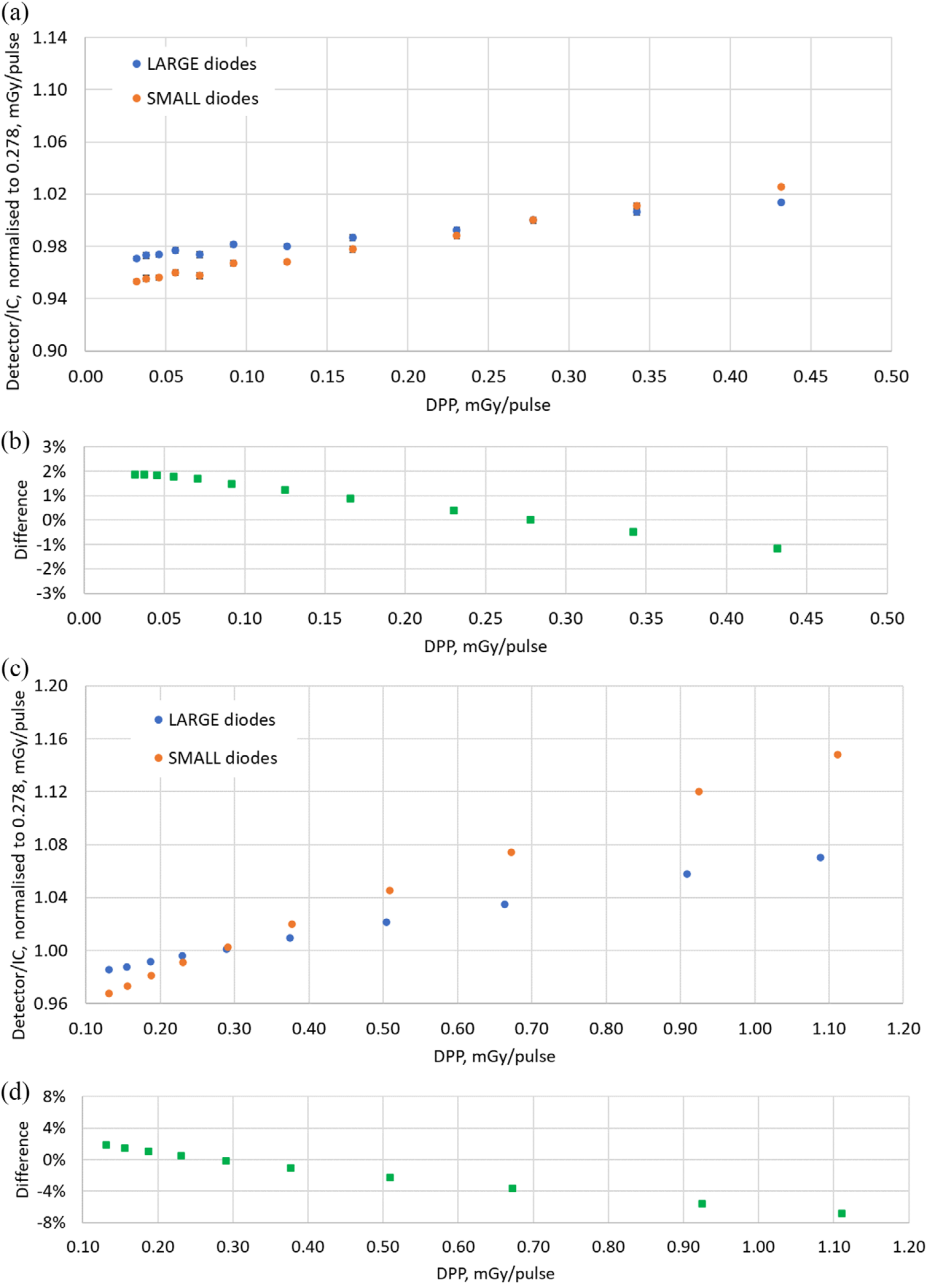
Dose‐per‐pulse (DPP) dependence of MP976 diodes relative to Farmer chamber (IC) readings and normalized to DPP of 0.278 mGy/pulse. The results are averaged among all diodes, either “large” or “small”. (a) results for the 6X beam; (b) difference between “large” and “small” for 6X; (c) results for the 10FFF beam; (b) difference between “large” and “small” for 10FFF.

Change of sensitivity due to variation in dose per pulse is depicted in Figure 8 (a‐b for 6X and c‐d for 10FFF). The results are extracted separately for “large” and “small” diodes and normalized to a DPP of 0.278 mGy/pulse. The error bars represent one standard deviation over three measurements. The MP976 diodes show a good agreement in dose‐per‐pulse dependence with commercially available single diodes used in arrays.^23^

### 3.4 Output factors

**FIGURE 9:**
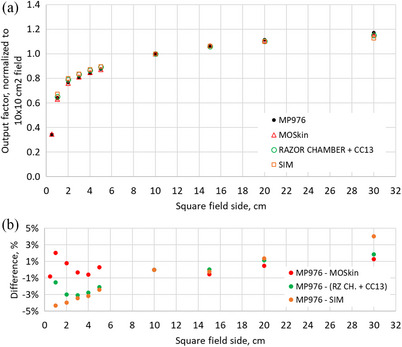
(a) Output factor (OF) study results comparing MP976 with MO*Skin*, LINAC commissioning data (Razor chamber + CC13), and Monte Carlo simulation of the OF; (b) the difference between the MP976 and the reference data.

Figure 9 shows the results for output factor measurements in comparison with MO*Skin* detector, LINAC commissioning data measured with CC13 and Razor chamber, and Monte Carlo simulations. The error bars represent one standard deviation over three measurements and lie within the marker size.

The resulting values for MP976 show a good agreement with MO*Skin* (within 2.0%) and LINAC commissioning data (within 3.1%) and acceptable agreement with Monte Carlo simulation (within 4.3%).

### 3.5 Angular dependence

**FIGURE 10:**
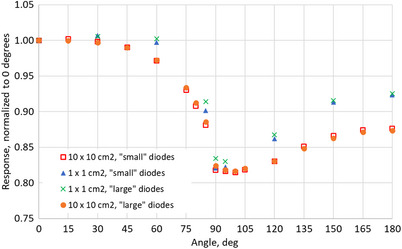
Angular dependence of MP976's “small” (average of four diodes) and “large” (average of two diodes) diodes irradiated with 10X beams of 10 × 10 cm^2^ and 1 × 1 cm^2^ field sizes. Error bars (standard deviation) do not exceed 0.12% (smaller than the marker size).

**FIGURE 11:**
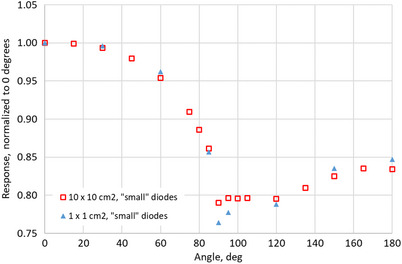
Angular dependence of MP976's “small” (average of four diodes) diodes irradiated with 6X beams of 10  × 10 cm^2^ and 1 × 1 cm^2^ field sizes. Error bars (standard deviation) do not exceed 0.12% (smaller than the marker size).

Results for the raw (not corrected) polar angular dependence of the MP976 detector for 10X and 6X are presented in Figures 10 and 11, respectively. The error bars represent one standard deviation over three measurements. The overall trend of the angular response of MP976 stays in good agreement with the previous studies of the MagicPlate detectors.^15^, ^24^, ^25^

For 10X beams, the angular response was measured for both “small” (central) and “large” (peripheral) diodes and showed no significant difference. The lowest relative response of 82% is attributed to 90° for the 1 × 1 cm^2^ field and to 100° for the 10 × 10 cm^2^ field. Overall, angular dependence for the two field sizes follows the same trend but has significant differences at all angles other than 0°–30° and 90°–95°.

As for the 6X measurements, the lowest relative response for the 1 × 1 cm^2^ field is 76%, and for the 10 × 10 cm^2^ is 79%, both at 90°. The results show a good agreement between both field sizes throughout all the angles, except for 90°–95°. This confirms the field‐size independence of the angle correction factors for 6X beams for angles up to 85°. Due to this fact and the absence of any significant difference between the “small” and “large” diodes in the 10X measurements, it was decided that separate measurements for the different diodes were not needed for 6X.

### 3.6 Depth dose profile

**FIGURE 12:**
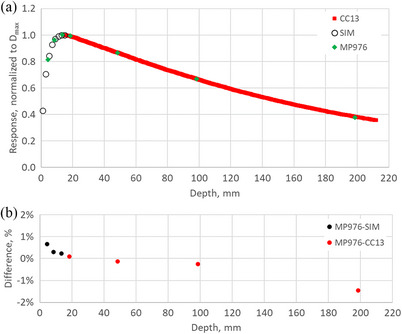
(a) Percent depth dose (PDD) profile for 6X beam measured with MP976 and compared to commissioning data measured with CC13 and to Monte Carlo simulation. Results are normalized to D_max_ = 15 mm, and the error bars (standard deviation) do not exceed 0.23% (smaller than the marker size). (b) difference between the MP976 results and the reference.

**FIGURE 13:**
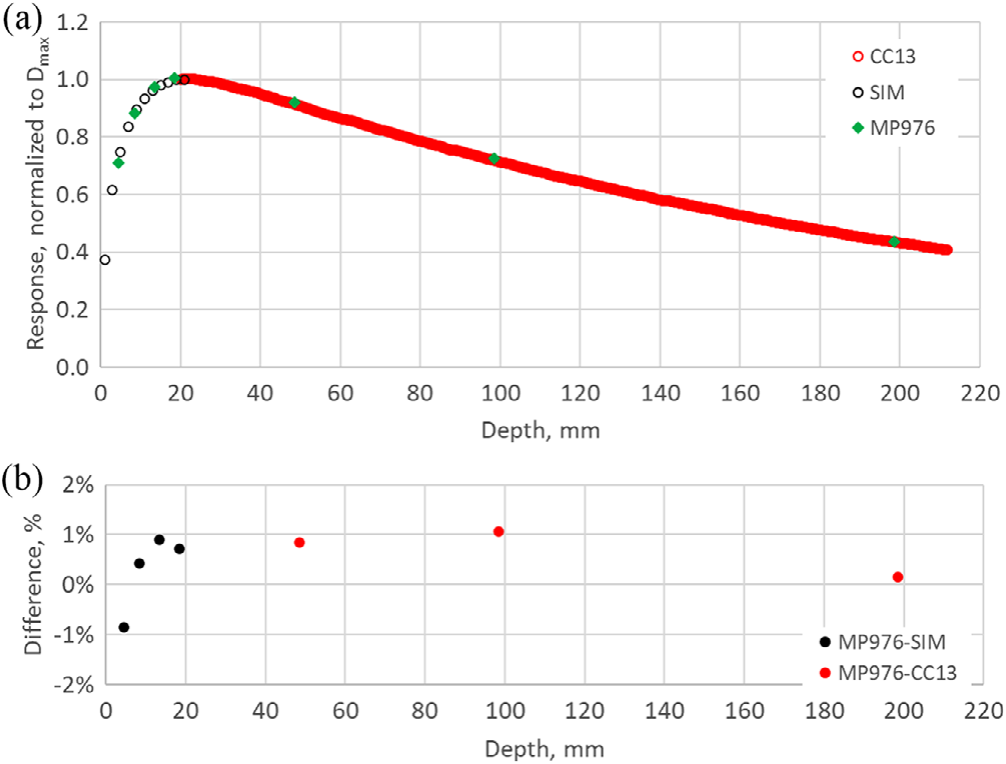
(a) Percent depth dose (PDD) profile for 10FFF beam measured with MP976 and compared to commissioning data measured with CC13 and to Monte Carlo simulation. Results are normalized to D_max_ = 20 mm, and the error bars (standard deviation) do not exceed 0.16% (smaller than the marker size). (b) difference between the MP976 results and the reference.

Percent depth dose (PDD) profiles for 6X and 10FFF beam qualities are depicted in Figures 12 and 13, respectively. The results are compared with the CC13 ionization chamber for depths beyond D_max_ and with Monte Carlo simulation for the build‐up region. The MP976 results represent the average response of the 4 central diodes corrected for dose‐per‐pulse dependence. The error bars represent one standard deviation over three measurements.

MP976 showed a very good agreement with the reference data with the difference in the majority of the points not exceeding 1% (maximum 1.46%).

### 3.7 Small field off‐axis profiles

**FIGURE 14:**
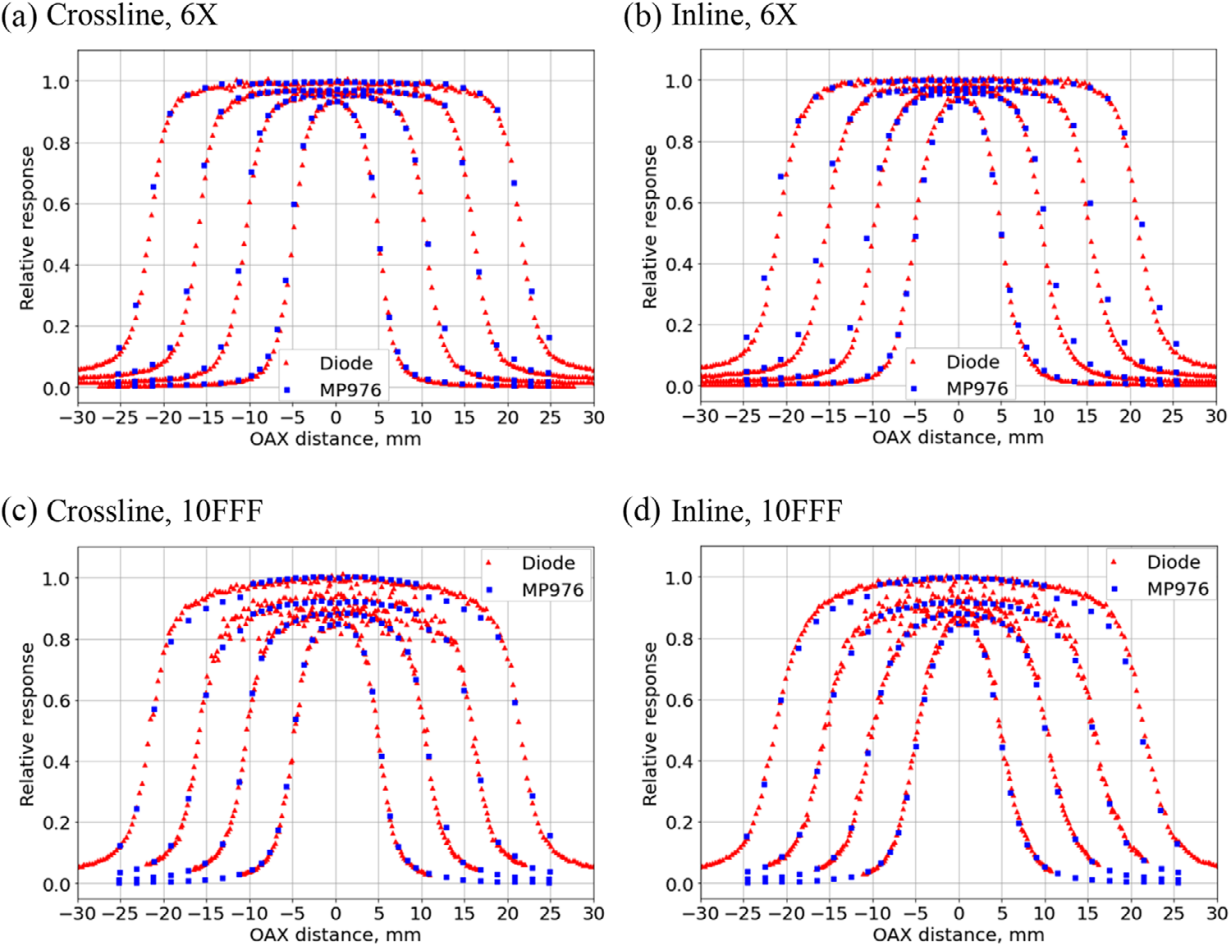
Off‐axis profile measurements for 6X and 10FFF beams of 1 × 1, 2 × 2, 3 × 3, and 4 × 4 cm^2^ jaws‐defined field sizes. The MP976 results are normalized to the LINAC commissioning data (EFD‐3G diode) at each beam size and the commissioning data is normalized to the CAX of the 4 × 4 cm^2^ beam. The error bars for the MP976 measurements do not exceed the marker size.

Figure 14 shows results for the 6X and 10FFF OAX profiles for field sizes of 1 × 1, 2 × 2, 3 × 3, and 4 × 4 cm^2^ defined by the LINAC jaws. The MP976 measurements are compared with the LINAC commissioning data measured with the EFD‐3G diode. The error bars represent one standard deviation over three measurements.

Tables 2, 3 present the quantitative comparison of the profiles measured using FWHM and penumbra 80%–20% criteria. The penumbra measurements with MP976 agreed with the reference within 0.5 mm for 6X and within 0.3 mm for 10FFF. FWHM values of the beam profiles matched the reference within 0.5 mm for both beam qualities.

**TABLE 2:** Crossline and inline OAX profile measurements for 6X beams.

Field size, cm^2^	MP976, mm	Diode, mm	MP976‐Diode difference
FWHM, crossline profile			
1 × 1	10.5	10.1	0.4 mm
2 × 2	21.5	21.1	0.4 mm
3 × 3	32.6	32.1	0.5 mm
4 × 4	43.7	43.2	0.5 mm
FWHM, inline profile			
1 × 1	10.3	10.5	−0.2 mm
2 × 2	21.1	19.9	1.2 mm
3 × 3	32.2	30.9	1.3 mm
4 × 4	43.3	42.1	1.2 mm
80%/20% for LHS‐RHS average penumbra, crossline profile			
1 × 1	2.7	2.7	0.0 mm
2 × 2	3.4	3.1	0.3 mm
3 × 3	3.5	3.3	0.2 mm
4 × 4	3.6	3.3	0.3 mm
80%/20% for LHS‐RHS average penumbra, inline profile			
1 × 1	3.4	3.1	0.3 mm
2 × 2	4.0	3.5	0.5 mm
3 × 3	4.2	3.7	0.5 mm
4 × 4	4.3	3.8	0.5 mm

Abbreviations: FWHM, full width at half maximum; LHS, left‐hand‐side; RHS, right‐hand‐side; OAX, off‐axis.

**TABLE 3:** Crossline and inline OAX profile measurements for 10FFF beams.

Field size, cm^2^	MP976, mm	Diode, mm	MP976‐Diode difference
FWHM, crossline profile			
1 × 1	10.5	10.3	0.2 mm
2 × 2	21.3	21.2	0.1 mm
3 × 3	32.1	32.3	−0.2 mm
4 × 4	43.1	43.0	0.1 mm
FWHM, inline profile			
1 × 1	10.2	10.7	−0.5 mm
2 × 2	20.8	21.1	−0.3 mm
3 × 3	31.7	31.7	0.0 mm
4 × 4	42.5	42.9	−0.4 mm
80%/20% for LHS‐RHS average penumbra, crossline profile			
1 × 1	2.8	3.0	−0.2 mm
2 × 2	3.7	3.6	0.1 mm
3 × 3	4.2	3.9	0.3 mm
4 × 4	4.6	4.6	0.0 mm
80%/20% for LHS‐RHS average penumbra, inline profile			
1 × 1	3.7	3.8	−0.1 mm
2 × 2	4.4	4.6	−0.2 mm
3 × 3	5.2	5.0	0.2 mm
4 × 4	5.6	5.5	0.1 mm

Abbreviations: FWHM, full width at half maximum; LHS, left‐hand‐side; RHS, right‐hand‐side; OAX, off‐axis.

It should be noticed that 6X inline profiles for 2 × 2, 3 × 3, and 4 × 4 cm^3^ field sizes had deviations of 1.2, 1.3, and 1.2 mm, respectively. Subsequent investigation of this inconsistency showed that the corresponding commissioning data available to the authors were taken before the LINAC went through a correction of 1 mm error in the Y‐jaw position. The 10FFF data was collected after the adjustment. Therefore, the FWHM estimations for the 6X commissioning data for 2 × 2, 3 × 3, and 4 × 4 cm^3^ fields should be considered unreliable.

## 4 DISCUSSION

After the priming, the slightly larger drop in sensitivity for the “small” diodes can be explained by the relatively larger contribution of laterally collected charge to the total response of the diodes in comparison with the “large” ones. To simplify, each diode's response can be represented as a sum of the charge collected from under the junction and the charge collected laterally from the region between the diodes. The p‐n junctions in MP976 are fabricated on a thin epitaxial layer (38 µm); it is assumed that the diffusion length stays much larger than this thickness even after the priming and hence the generated charge is fully collected from under the junction. Due to their size and pitch, the lateral diffusion part is more pronounced for the “small” diodes making them more sensitive to its variation. This gives a larger effect of the charge carrier diffusion length shortening with accumulated dose. It is worth noting that the ratio of the responses of “large” diodes to “small” ones (3.26 ± 0.34 before priming and 3.95 ± 0.10 after 10 kGy) is lower than the ratio of the areas under the corresponding junctions (9.00) which points to a significant contribution of the laterally collected charge to the overall response.

A similar approach can be used to explain the difference in the dose‐per‐pulse dependence between the “large” and “small” diodes, which amounted to (3.0 ± 0.3)% for 6X between 0.032 and 0.432 mGy/pulse and (8.4 ± 0.2)% for 10FFF between 0.131 and 1.111 mGy/pulse (Figure 8b–d). Since diffusion length is a function of an injection rate (in our case of pulsed LINAC radiation, it is dose per pulse), the variation in DPP affected the lateral charge collection. This effect may influence the charge‐sharing between diodes and the effective area of the charge collection causing the difference in DPP dependence among “large” and “small” diodes.

**FIGURE 15:**
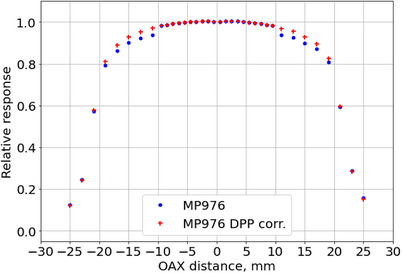
Comparison of the 10FFF 4 × 4 cm^2^ crossline profile measured by MP976 with and without correction for the dose‐per‐pulse dependence. Equalization of the detector's diodes was performed using a flattened 10 MV 20 × 20 cm^2^ beam (defined at the isocenter) measured at a depth of 10 cm and SSD of 100 cm. SSD, source‐to‐surface distance.

This effect can potentially cause deviations in the estimation of dose distributions, especially if the DPPs of the beams used for equalization and calibration of the detector significantly differ from those of the measured beams. As an example, Figure 15 shows the profile of the 4 × 4 cm^2^ 10FFF beam from Section 3.7. with and without DPP correction. Equalization of the detector diodes was performed using a flattened 6 MV beam which has lower DPP compared to the measured 10FFF field, therefore a clear “step” in the response between the outermost “small” diode and the innermost “large” diode can be seen. Correction for the DPP dependence fully eliminated the effect. Therefore, the detector should be equalized and calibrated with the beam quality as close to the measured one. Further studies will be conducted to determine whether this phenomenon has any significant impact on the overall performance of the detector at clinically relevant dose distributions.

The good agreement of the output factors shows that the detector packaging incorporating a small air gap on top of the sensitive volumes makes MP976 field size independent for the clinically relevant 6X beams. Despite the relatively large volume of silicon around the sensitive volumes compared to single‐diode dosimeters, the results showed an excellent match with the reference for the depth dose measurements.

The angular response of MP976 can be attributed to the anisotropy of the materials surrounding the sensitive volumes (SVs) (mainly by the silicon layer and PCB below the SVs and the air gap on top of the SVs) that leads to angle‐dependent perturbation of the photon and secondary electron fields. Two competing processes are happening in the materials around SVs: attenuation on one hand, and increased production of the electrons due to higher density (dose enhancement) on the other.^25^ These processes are strongly dependent on the density of the materials and the energy of photons and electrons. Since for 10 MV beams, both photon and secondary electrons energies are harder than for 6 MV, the combined effect of the stated processes creates a larger difference between the 1 × 1 cm^2^ and 10 × 10 cm^2^ fields, especially for angles 120°–180°. The fact that the relative response at 90°–180° (behind the detector) for both 6 and 10 MV is asymmetrical and does not recover back to 100% is due to the larger attenuation of the beam by the PCB and silicon substrate layers. Virtually any semiconductor array exhibits angular dependence, the effect can be corrected by monitoring the gantry angle with an inclinometer as it is currently done in commercially available 2D array systems and was shown elsewhere for another detector of the MagicPlate family.^6^, ^10^

The MP976 measurements of off‐axis small field profiles showed very good agreement with the reference in the 80%/20% penumbra criterion. This parameter is considered important for verifying steep‐gradient dose distributions specific to highly conformal radiotherapy modalities delivering high doses close to critical organs, like SABR of spinal metastases where distance‐to‐agreement criteria is set at 1 mm. The monolithic pixelated silicon detector technology used in MP976, combined with highly sensitive readout electronics, allows for the fabrication of very small sensitive volumes and therefore minimizes dose volume averaging, which significantly impacts penumbra measurements.

## 5 CONCLUSION

A new 2D high temporal and spatial resolution monolithic pixelated semiconductor detector—MagicPlate‐976 (MP976)—was introduced and comprehensively characterized for small field dosimetry under clinical Megavoltage x‐ray beams. Compared to its predecessors, MP976 is fabricated on an *n*‐type 38 µm epitaxial layer placed on a low resistivity silicon substrate, has a larger array area, and does not require an external trigger pulse from a LINAC. The introduced non‐uniform arrangement of diodes allows for higher spatial resolution for very small field sizes of less than 2 × 2 cm^2^ while keeping a reasonable number of readout channels and compact electronics. After 10 kGy priming, the MP976 detector showed a low sensitivity change with dose allowing for measuring high‐dose clinical plans like spinal SABR. Due to its high sampling rate (up to 5 kHz), the system has no dependence on pulse rate (nominal dose rate in terms of MU/min) under clinical LINAC beams. The dose‐per‐pulse dependence lies within the values of commercial diodes used for 2D arrays. Despite a relatively large area of the silicon substrate, the detector showed a very good match in the depth‐dose measurements (dose‐per‐pulse dependence corrected) and reasonable angular dependence. Output factor and lateral profile measurements showed that the MP976 system is suitable for dosimetry in small fields and high dose gradients providing 1 mm spatial resolution in the central region and requiring no field size correction down to 0.5 × 0.5 cm^2^ field.

The shown characteristics and performance of the MagicPlate‐976 detector system open new opportunities for trigger‐less, film‐less, real‐time, and time‐resolved verification and error identification for complex stereotactic treatment plans. There are ongoing studies in cooperation with the Illawarra Cancer Care Centre (Illawarra Shoalhaven Local Health District, Wollongong, New South Wales, Australia) and the Peter MacCallum Cancer Centre (Melbourne, Victoria, Australia) to investigate MP976's performance for real‐time and time‐resolved verification of spinal SABR treatment plans directly in the axial plane and for fast end‐to‐end testing of dose placement for stereotactic modalities using hidden targets with different density ratio.

## AUTHOR CONTRIBUTIONS

**Ilia Filipev**: Data curation; formal analysis; investigation; methodology; project administration; software; visualization; writing—original draft. **Saree Alnaghy**: Data curation; formal analysis; investigation; methodology; software; writing—review & editing. **Martin Carolan**: Conceptualization; investigation; validation; resources; writing—review & editing. **Jason Paino**: Data curation; visualization; writing—review & editing. **Marco Petasecca**: Conceptualization; investigation; methodology; validation; writing—review & editing. **Dean Cutajar**: Conceptualization; investigation. **Joel Poder**: investigation; resources; validation; writing—review & editing. **Justin B. Davies**: Investigation; resources. **Bradley M. Oborn**: Data curation; software. **Nicholas Hardcastle**: Formal analysis; investigation; methodology; validation. **Susanna Guatelli**: Conceptualization; supervision; writing—review & editing. **Michael Lerch**: Conceptualization; writing—review & editing. **Tomas Kron**: Conceptualization; methodology; resources; supervision; validation; writing—review & editing. **Anatoly Rosenfeld**: Conceptualization; methodology; resources; supervision; validation; writing—review & editing.

## CONFLICT OF INTEREST STATEMENT

The authors declare no conflicts of interest.
